# Intrathecal Adjuvant Midazolam Versus Fentanyl With Hyperbaric Bupivacaine for Post-operative Analgesia in Women Undergoing Total Abdominal Hysterectomy

**DOI:** 10.7759/cureus.40565

**Published:** 2023-06-17

**Authors:** Aishwarya Nayak, Sanjot Ninave, Surekha Tayade, Harshal Tayade

**Affiliations:** 1 Department of Anaesthesiology, Jawaharlal Nehru Medical College, Datta Meghe Institute of Higher Education and Research, Wardha, IND; 2 Department of Obstetrics and Gynaecology, Jawaharlal Nehru Medical College, Datta Meghe Institute of Higher Education and Research, Wardha, IND; 3 Department of Surgery, Mahatma Gandhi Institute of Medical Sciences, Wardha, IND

**Keywords:** postoperative analgesia, postoperative pain management, adjuvants to spinal anesthesia, intrathecal adjuvants for post operative analgesia, adjuvants to subarachnoid block

## Abstract

Background

The administration of adequate analgesia post-operatively has been associated with fewer cardiopulmonary complications, lower morbidity and mortality, lower healthcare costs, and higher patient satisfaction. One of the most effective ways to raise the standard of healthcare would be standardization of multimodal analgesia with enhanced recovery after surgery. Adjuncts to spinal anesthetists can achieve a better post-operative pain relief with less doses of rescue analgesia.

Methods

This was a prospective, randomized trial conducted on 60 women undergoing abdominal hysterectomy under spinal anesthesia. We evaluated the impact of adding 0.5 mL (2.5 mg) of intrathecal midazolam versus 25 mcg of intrathecal fentanyl (0.5 mL) with 2.5 mL injection of 0.5% bupivacaine (hyperbaric) (12.5 mg). The outcomes were prolongation of analgesia in the post-operative period, onset and duration of sensory and motor blockade, stable hemodynamics, and any adverse reactions to the study drugs.

Results

The two groups, group M (midazolam + hyperbaric bupivacaine) and group F (fentanyl + hyperbaric bupivacaine), had similar distribution for age, weight, and type and duration of surgical procedure. Both groups had stable vital parameters and experienced a similar onset of sensory and motor blockade. Intraoperative modified Ramsay sedation score was better in group M in comparison to group F. However, mean of elapsed time of two-segment regression of sensory block, from T12 to L1 level, and mean time to regression, from Bromage score 3 to 2, were longer in group F. Group F also had a better visual analogue scale (VAS) score in the post-operative period than group M, and group F experienced a longer average post-operative analgesic duration (lasting for 367.73 minutes) as compared to group M (lasting for 254.9 minutes), having a difference that was of statistical significance (p < 0.001). No substantial adverse reactions were seen in either group.

Conclusion

The duration of post-operative analgesia is significantly prolonged when 25 mcg of adjuvant intrathecal fentanyl is used with 0.5% bupivacaine (hyperbaric) as compared to intrathecal midazolam 0.5 mL (2.5 mg) in women undergoing abdominal hysterectomy under spinal anesthesia. Both fentanyl and midazolam have minimal adverse reactions and are safe to use as adjuvants to 0.5% of bupivacaine (hyperbaric) in surgeries conducted in the lower abdomen.

## Introduction

There have been several researches on the individual potencies of fentanyl and midazolam when used as adjuvants to bupivacaine (hyperbaric), but only a few have been conducted to compare them. Although total abdominal hysterectomy is a common gynecological procedure performed on women for various indications, post-operative pain relief with intrathecal adjuvants has not been widely studied in women undergoing this surgery [1,2].

A great amount of attention and importance has been placed on understanding the synergistic effect of various drugs in the field of pain relief [3]. By administering intrathecal drugs as adjuvants targeted at a variety of spinal-cord receptors, a superior quality of prolonged analgesic effect is achievable. Dose reductions may be advantageous in avoiding dose-related side effects of these drugs.

Another method for improving pain relief is to simultaneously target multiple receptor sites of the spinal cord [3]. Even with high sensory block, bupivacaine is insufficient to provide adequate post-operative analgesia and necessitates a high dose of rescue analgesia to reduce post-operative pain. It is a matter of moral obligation for an anesthesiologist to give the patient a post-operative phase that is both safe and free of pain by employing different medications, techniques, and combinations therein in order to facilitate the early ambulation and discharge of the patient. To hasten the speed of onset, enhance the quality, and broaden the scope of subarachnoid block, numerous additive agents to neuraxial anesthetics are available. These include opioid drugs such as fentanyl, morphine, buprenorphine, n-methyl-d-aspartate antagonists (e.g., ketamine), vasoconstrictor agents (e.g., adrenaline), gamma-aminobutyric acid (GABA) receptor agonists (e.g., midazolam), alpha-2-adrenoceptor agonist (e.g., clonidine and dexmedetomidine), and cholinergic agents (e.g., neostigmine and sodium bicarbonate). When an adjuvant is added to a local anesthetic agent for subarachnoid blockage, the effect is amplified and post-operative analgesia is prolonged. It helps reduce the need for post-operative analgesics and dose of bupivacaine. In addition, it maintains the hemodynamic stability of the patient [4]. Fentanyl is an opioid receptor agonist that is synthetically prepared and is a type of phenyl piperidine derivative that is extremely potent due to its high lipophilicity [5]. Because of its rapid onset and narrowed time of action, with minimalistic cephalad spread, its use as an adjuvant to subarachnoid anesthesia is preferred. In combination with lowered dose bupivacaine, fentanyl improves the anesthetic effect during surgery and also increases the reliability of block [5]. However, side effects such as nauseous feel, vomiting, pruritus, depressed respiration, tremors, and retention of urine necessitate further research into alternative adjuvants.

In the quest for a novel and safe adjuvant to a local anesthetic agent, scientists discovered that benzodiazepines (BZDs) have a “segmental nociception blockade” with no adverse effects on the cardiovascular, respiratory, or nervous system. One such BZD is midazolam. BZD receptors, which are linked to GABA receptors, can be found in the spinal cord and the central nervous system. Midazolam given intrathecally with bupivacaine raises pain thresholds by binding to BZD receptor complexes in the spinal cord [5,6]. It is known to induce antinociception and enhance the effect of local anesthesia when administered in neuraxial blockage without causing significant side effects [7].

The current study compares the effect of adjuvant midazolam versus fentanyl provided intrathecally with injection bupivacaine (hyperbaric) for analgesic action in the immediate period after an operative procedure in women who are being operated for hysterectomy by abdominal route. The overarching goal is to guide clinical practice by commenting on comparative efficiency and adverse reactions.

## Materials and methods

This was a randomized trial carried out in the anesthesiology unit of a rural tertiary care hospital based in central India. Approval for the study was obtained from the institutional review board. Consenting women, aged 35-60 years, with a weight ranging from 40 to 75 kg and having a physical status of ASA (American Society of Anesthesiologists) I and II categories and Mallampati classification (MPC) of first grade, and undergoing total abdominal hysterectomy under subarachnoid block anesthesia were recruited as the trial participants. -The study excluded women with higher ASA and MPC status or those having pre-operative hematological, hepatic, renal, cardiac, respiratory, neurological, and musculoskeletal disease, abnormal coagulation profile, history of thromboembolic episode, increased intracranial pressure, spinal deformity, and other usual contraindication to subarachnoid block. Sixty consecutive women who met the study eligibility criteria were recruited and randomly assigned to two groups using digitally generated random number tables. The two groups are detailed in Table 1.

**Table 1:** Description of two groups in which randomization of participants was done

Group		Total Volume
Group M (30 study participants)	2.5 mL of injection-grade (heavy) bupivacaine ( 12.5 mg) + 0.5 ml of injection-grade midazolam (2.5 mg)	3 mL
Group F (30 study participants)	2.5 mL of injection-grade (heavy) bupivacaine (12.5 mg) + 0.5 mL of injection-grade fentanyl (25 mcg)	3 mL

The procedure of study is explained in Table 2.

**Table 2:** Methodology of study ECG, electrocardiography; DBP, diastolic blood pressure; HR, heart rate; IV, intravenous; MAP, mean arterial pressure; NIBP, non-invasive blood pressure; RR, respiratory rate; SBP, systolic blood pressure; VAS, visual analogue scale

Study Procedure
The patient was counselled, an explanation was provided about the anesthesia procedure including VAS, and informed consent was sought. Age (in years), weight (in kilograms), ASA grade, and surgical indication were all recorded on the predesigned performa.
After arrival in the operation theater, a multipara monitor was attached to the patient to record vital parameters such as HR, RR, SpO_2_, NIBP, and ECG.
An 18G IV cannula was inserted, and preload IV fluid (ringer lactate solution) at the rate of 10 mL/kg was provided.
Baseline parameters such as HR, RR, SBP, DBP, MAP, and SpO_2_ were measured, and a continuous visual display of a 5-lead ECG was affirmed.
All study participants were premedicated with injection ondansetron 75 mcg/kg intravenously over 10 minutes prior to anesthesia.
To monitor urine output, Foley's catheter was inserted per urethral.
With due asepsis and antisepsis, spinal anesthesia was given in the left-lateral/sitting position in the midline with 25- to 26-gauge Quincke’s spinal needle in L3-4/L4-5 intervertebral space, and a designated drug was given slowly after assuring free flow of cerebrospinal fluid.
The patient was turned to supine position, and a pillow was placed below the shoulder
The pinprick method with a blunt-tipped needle was used to test for sensory block. The modified Bromage scale was used to evaluate the quality of motor block [4], and operative procedure was allowed.
The following parameters were recorded intraoperatively:
1. Time of onset of sensory-motor block
2. Time taken and the maximum level of sensory and motor block
3. Two-segment regression time
4. Sedation level was assessed using the Modified Ramsay sedation scale at 5, 10, and 15 minutes and then every 15 minutes until surgery was completed, with the highest sedation score recorded.
5. HR, RR, SBP, DBP, MAP, and SpO_2_ were noted at 0 hour, then every 2 minutes until 10 minutes, then every 15 minutes until 30 minutes, and, finally, half hourly until surgery was completed.
6. Any adverse reaction such anxiety, nauseous feel, vomiting, bradycardia/tachycardia, hypotension, shivering, pruritic rash, etc.
The following parameters were recorded post-operatively:
1. Pain intensity and quality by a VAS scale at the end of operative procedure and after transfer to the recovery room.
2. Time to first rescue analgesia
3. Adverse reactions
Management of side effects:
1. Any side effects observed following intrathecal drug administration were documented and treated appropriately
2. In the event of HR falling to 60 beats per minute (bradycardia) or 30% less than the baseline, patients were given IV injection of glycopyrrolate 0.2 mg.
3. In the event of hypotension (a drop in blood pressure below 20% of baseline), mephentermine was administered intravenously in titrated boluses.
4. IV fluids were administered based on the patient’s weight (2 mL/kg/hr), and maintenance fluid was administered after calculating urine output per hour. Intraoperative blood loss was calculated as follows: 1 fully soaked mop = 100 mL, 1 partially soaked mop = 50 mL, 1 fully soaked gauze piece = 10 mL, 1 partially soaked gauze piece = 5 mL, and replaced appropriately.
5. Injection ondansetron 4 mg was giving to control nausea and vomiting. Injection tramadol 50 mg was given for shivers, and pruritic rash/allergy was treated with IV injection hydrocortisone 100 mg and IV injection pheniramine maleate 45.5 mg.
6. After the surgery was completed, the patient was shifted to the post-operative ward, with intermediate 2 hours monitoring in a high-dependency unit. Monitoring was performed every hourly for the first 6 hours and then every 2 hours over the day. When the patient complained of pain, and a VAS score of 4 or higher was obtained, rescue analgesia was administered with IV paracetamol (15 mg/kg).

Statistical analysis

Using appropriate statistical tests, the efficacy of the study drugs for post-operative analgesia and adverse reactions were analyzed, and comparison was made between the two groups. The data were presented in the form of percentages, proportions, means, and standard deviations. For the analysis of continuous variables, analysis-of-variance test was used, while two-tailed Fisher’s test and the chi-square tests were used for analyzing categorical information. The unpaired Student’s t-test compared the data sets. A p-value of less than 0.05 was considered statistically significant.

## Results

The two groups, group M (midazolam + hyperbaric bupivacaine) and group F (fentanyl + hyperbaric bupivacaine), had similar distribution for age, weight, and type and duration of operative procedure. There was no statistically significant difference in the average age, weight, and mean time taken for operative procedure, in both groups, as similar values were found (p > 0.5) (Table 3).

**Table 3:** Mean age, weight, and duration of surgery of study participants NS, not significant

Parameter	Group M	Group F	P-value
Mean age (years)	44.86±7.40	43.26±6.43	0.37 ( NS)
Mean weight (kilograms)	59±5.53	58 ±6.54	0.52 ( NS)
Mean time taken for surgery (minutes)	168.56±11.44	170.06± 9.81	0.58 ( NS)

The distribution of cases according to diagnosis was comparable in both the groups, and the difference had no statistical significance (p = 0.98) (Table 4).

**Table 4:** Distribution of study participants according to diagnosis

Diagnosis	Group M, N (%)	Group F, N (%)	Total, N (%)
Abnormal uterine bleeding	12 (40)	11 (36.7)	23 (38.3)
Chronic cervicitis	3 (10)	4 (13.3)	7 (11.7)
Fibroid uterus	7 (23.3)	6 (20)	13 (21.3)
Menorrhagia	5 (16.7)	5 (16.7)	10 (16.7)
Postmenopausal bleeding	3 (10)	4 (13.3)	7 (11.7)
Total	30 (100)	30 (100)	60 (100)

**Figure 1:**
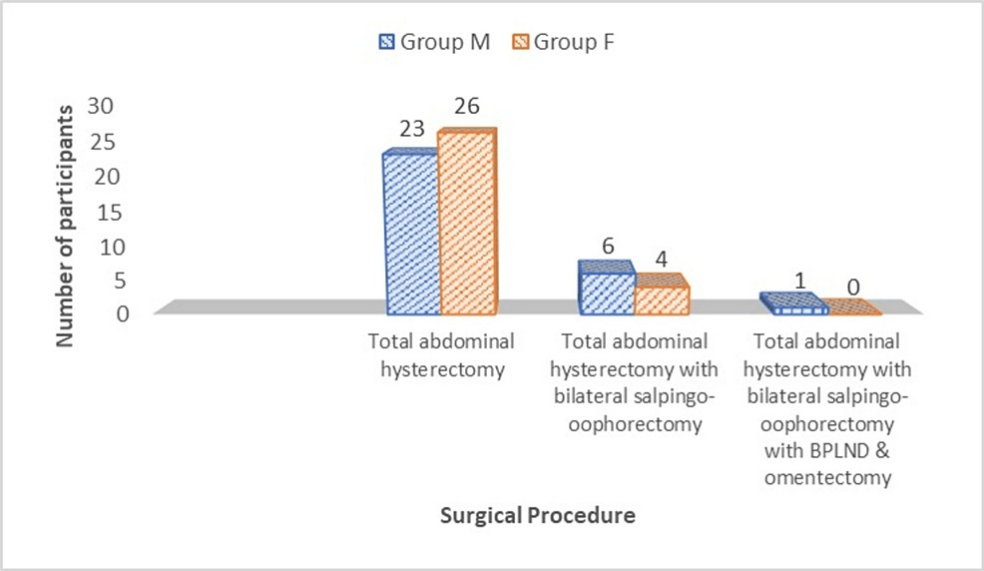
Distribution of study participants according to the surgery performed

Similar kind of surgery was performed in both the groups, and the difference had no statistical significance (p = 0.45) (Figure 1).

**Figure 2:**
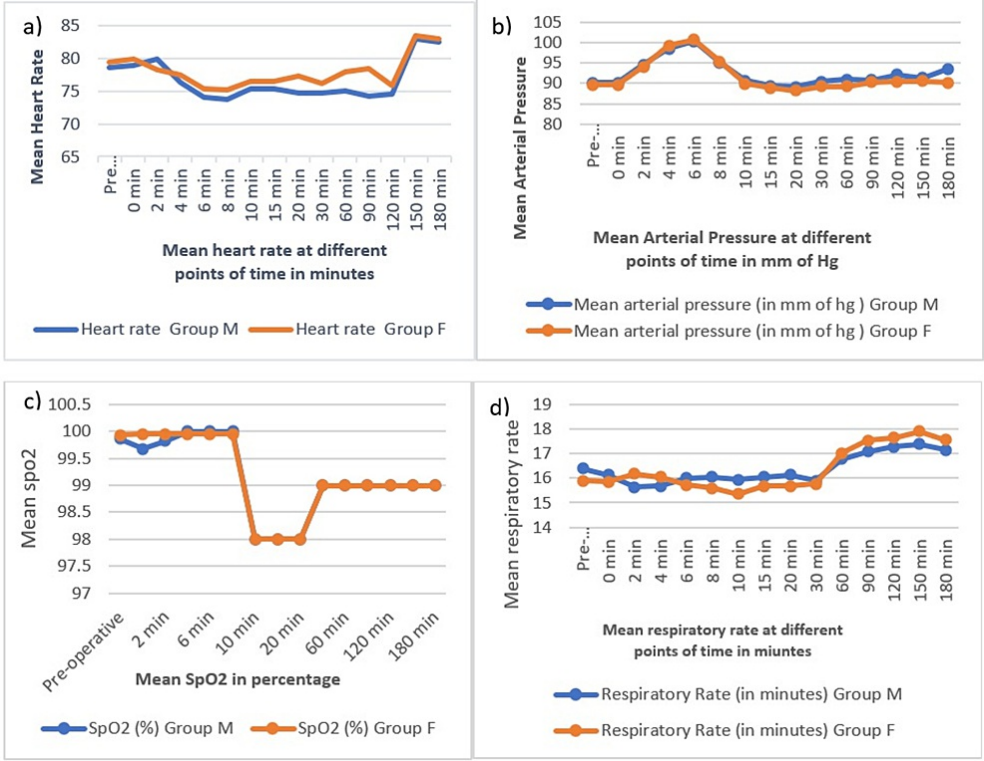
Mean heart rate, systolic blood pressure, SpO2, and respiratory rate in the two groups (a) Mean heart rate. (b) Mean arterial pressure. (c) Mean SPO_2_. (d) Mean respiratory rate.

Mean heart rate (HR) at various points of time, the mean arterial pressure (MAP), respiratory rate (RR), and oxygen saturation (SpO_2_) had close similarity across the two groups, and no statistical difference was found (p > 0.05) (Figure 2).

**Figure 3:**
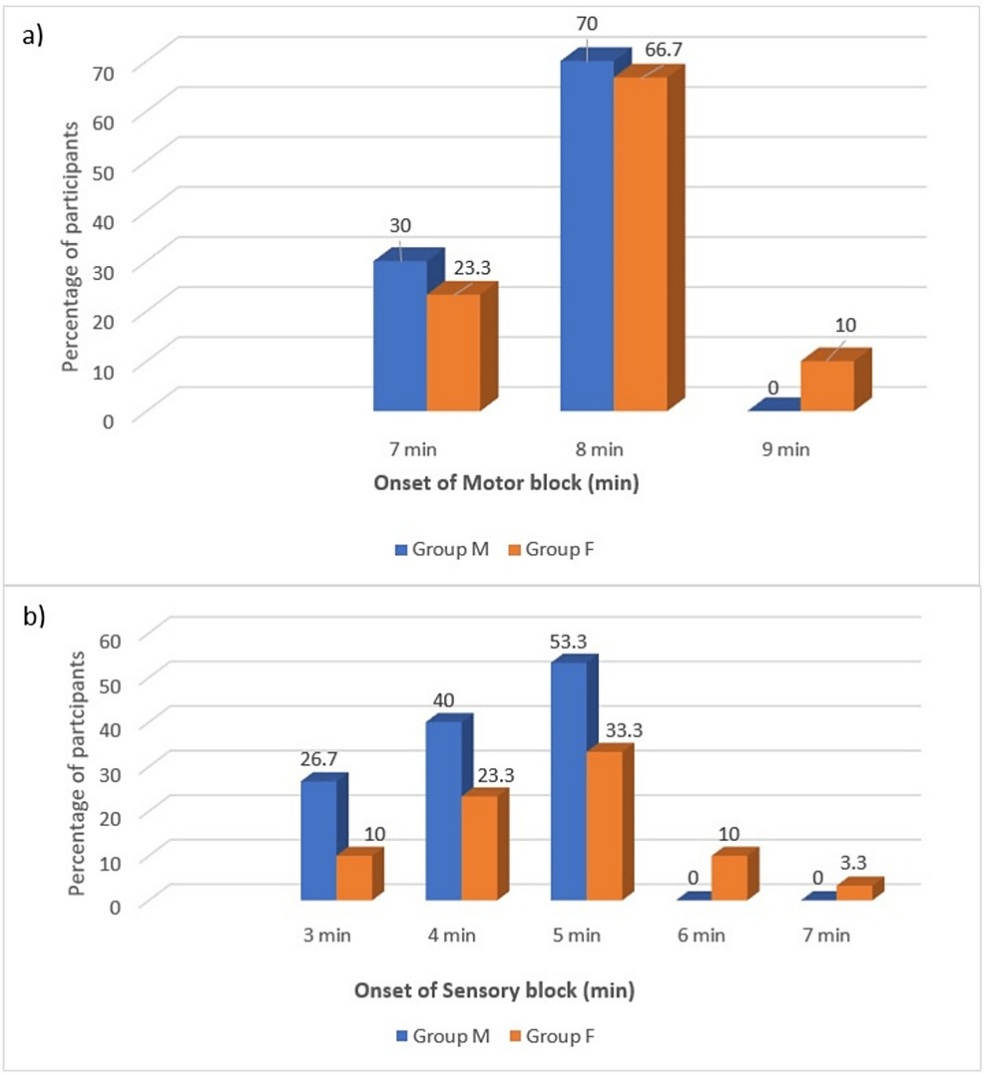
Onset of sensory and motor blockade in the two groups (a) Onset of motor block. (b) Onset of sensory block.

There was no statistical difference across the two groups in terms of onset of sensory block and motor block (p = 0.06 and 0.19, respectively) (Figure 3).

**Figure 4:**
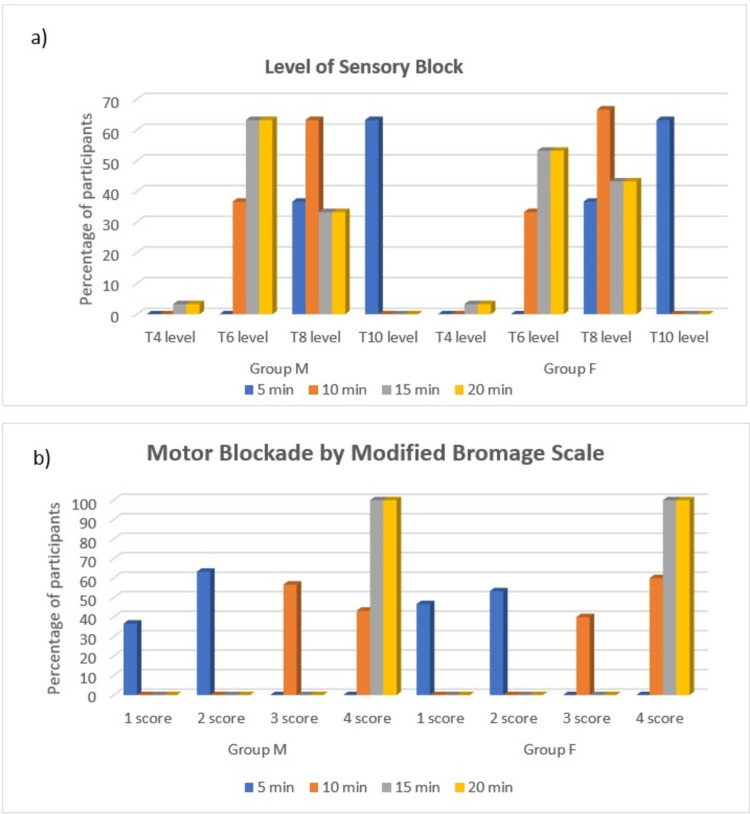
Level of sensory blockade and motor blockade by the modified Bromage scale (a) Level of sensory block. (b) Motor blockade by the modified Bromage scale.

There was no statistical difference across the two groups in terms of the level of sensory block, attained at different time points, and motor block by modified Bromage scale as values were comparable (p > 0.5) (Figure 4).

There was statistical difference in terms of the intraoperative modified Ramsay sedation score, as it was better in group M as compared to group F (p < 0.001) (Table 5).

**Table 5:** Intraoperative modified Ramsay sedation score

Intraoperative modified Ramsay sedation score	Group M, N (%)	Group F, N (%)	Total, N (%)
0	0	0	0
1	0	1 (3.3)	1 (1.6)
2	12 (40)	27 (90)	39 (65)
3	10 (33.3)	2 (6.7)	12 (20)
4	8 (26.7)	0	8 (13.3)
5	0	0	0
6	0	0	0
7	0	0	0
8	0	0	0
Total	30 (100)	30 (100)	60 (100)

The mean time duration for two-segment regression of sensory blockage, from T12 level to L1 level, was higher in group F as compared to group M, and the difference had statistical significance (p < 0.001) (Table 6).

**Table 6:** Mean time for two-segment regression of sensory block

Time points for regression of sensory block	Group M	Group F	P-value
At T12	205.3±6 min	184.83±6 min	<0.001
At L1	227.96±6 min	213.1±6 min	
2 segment regression from T12 to L1	22.66±6 min	28.27±6 min	

The mean time to regression from Bromage 3 to Bromage 2 scores was longer in group F compared to group M, and the difference had statistical significance (p < 0.001) (Table 7).

**Table 7:** Time to regression of motor block as per the modified Bromage scale score

Score according to the modified Bromage scale	Group M	Group F	P-value
Bromage 3	151.7±5.6 min	170.16±6 min	<0.001
Bromage 2	175.7±8.6 min	198.4±7 min	
Time to regression from Bromage 3 to Bromage 2 score	24±3 min	28 ± 1 min	

Two (6.66%) study participants had hypotension and two (6.66%) had bradycardia in group M, whereas one (3.33%) each had bradycardia, pruritis, and nausea, and two (6.66%) had hypotension in group F. This difference in adverse effects was not of statistical significance (p = 0.56) (Table 8).

**Table 8:** Side effects observed in both groups

Post-operative complications	Group M	Group F	P-value
Nausea	0	1 (3.33%)	0.56
Hypotension	2 (6.66%)	2 (6.66 %)	
Bradycardia	2 (6.66%)	1 (3.33)	
Pruritis	0	1 (3.33%)	

Group F had a better visual analogue scale (VAS) score in the post-operative period than group M (p < 0.001), and the mean duration of post-operative analgesia was greater in group F (lasting for 367.73 minutes) as compared to group M (lasting for 254.9 minutes), and the difference had statistical significance (p < 0.001) (Table 9).

**Table 9:** Postoperative VAS score and post-operative analgesia in both groups VAS, visual analogue scale

Postoperative VAS score	Group M	Group F	Total	P value
0	0	10 (33.3 %)	10 (16.7 %)	< 0.001
1	9 (30 %)	12 (40 %)	21 (35 %)	
2	13 (43.3 %)	8 (26.7 %)	21 (35 %)	
3	8 (26.7 %)	0	8 (13.3 %)	
Total	30 (100 %)	30 (100 %)	60 (100 %)	
Postoperative analgesia in minutes				
Group M		Group F		P-value
254.9 ± 9.39		367.73 ± 16.59		<0.001

## Discussion

In the present study, the effect of adding 0.5 mL of midazolam (2.5 mg) intrathecally versus Fentanyl 0.5 mL (25 mcg) with 0.5% of 2.5 mL of bupivacaine (hyperbaric) (12.5 mg) was compared to evaluate the prolongation of post-operative analgesic effect, onset of sensory-motor block, stability of hemodynamics, and any adverse reactions with the study drugs. The research subjects in both groups were similar in demographics and were hemodynamically stable with comparable vital factors such as HR, blood pressure (BP), RR, and SpO_2_. Ambuj et al. [8] in their study noted that the average values of HR and MAP, in the first 60 minutes following administration of sub-arachnoid anesthesia and in the first 60 minutes post-surgery did not alter with much significance between the two groups of midazolam + bupivacaine and fentanyl + bupivacaine. Panwar and Chittora [9] also found no clinically relevant changes in BP, HR, SpO_2_, and respiratory function after addition of fentanyl or midazolam to intrathecal ropivacaine. Similar findings were noted by Bhure et al. [10] when analyzing and contrasting fentanyl and midazolam as additives to 0.5% bupivacaine (hyperbaric). Mehta et al. [11] reported that BZDs cause a segmental block of nociceptives without having any negative affection of the heart, lungs, or nervous system. Findings of the present study are also corroborated by Yektas [12], Mehta et al [11], and Modir et al. [13]. Both fentanyl and midazolam do not affect the vital parameters exceptionally when given intrathecally as adjuvants; however, preloading with adequate fluids should be assured.

The time for onset of sensory blockade was faster in group M; however, overall, no statistical significance was found for onset of sensory blockade across the 2 groups (p = 0.06). Modir et al. [13] reported that midazolam shows a shorter time to onset of sensory blockade after spinal anesthetic (5.20±0.72), whereas fentanyl took 8.42±0.65 minutes. Similar findings of early sensory blockade with intrathecal midazolam have been reported by Mehta et al. [11], Panwar and Chittora [9], and Yektas [12]. In similarity, finding of early onset of sensory blockade was observed by Codero et al. [14] when midazolam was co-administered with bupivacaine. 

There was no meaningful dissimilarity in the onset of motor blockade in both the groups (p = 0.19), and similar findings have been reported by Mehta et al. [11], Panwar et al. [9], and Yektas [12].

Motor blockade at different points in times after the procedure was comparable in both the groups, with similar observations by Mehta et al. [11], Panwar et al. [9], and Yektas [12]. Median sensory-motor blockade achieved by fentanyl and midazolam as adjuvants remain similar, as the opioid receptors are as abundant as GABA receptors in the spinal cord.

The modified Ramsay sedation score was better in group M than group F (p < 0.001). Bhure et al. [10] found that six (20%) of 30 patients had sedation with additive intrathecal midazolam in comparison to bupivacaine. The findings of the present study are corroborated by Panwar and Chittora [9] and Tiwary and Punetha [15].

The two-segment regression was slower with statistical significance in group F than group M (p < 0.001), with similar findings by Ambuj et al. [8], Suraj et al. [16], Panwar and Chittora [9], and Parmar et al. [17]. Slower regression of fentanyl helps in the prolongation of post-operative analgesia.

The mean time duration for regression of motor blockade was meaningfully higher in group F in comparison to group M (p < 0.001). Ambuj et al. [8], Sawhney et al. [18], Parmar et al. [17], Panwar and Chittora [9], and Suraj et al [16] reported similar findings.

No statistical significant difference was found with respect to adverse reactions in both the groups. Also, none of the study participants developed any complications such as respiratory depression, tremors, and shivering, or any neurological deficit or post-dural puncture headache. This may be because a 25-gauge spinal needle was used, and study subjects were preloaded adequately before induction of subarachnoid block.

The most frequently reported adverse events with intrathecal midazolam and fentanyl as additive agents were bradycardia (10 % vs 3.33 %) and hypotension (6.66% vs 9.99%). Hypotension was not statistically significant in the present study as all study subjects were provided preload fluids of 10 mL/kg (Ringer’s lactate) before spinal block to prevent hypotension as per the conventional practice.

The mechanisms causing nausea are very intricate. There is activation of mu opioid receptors found in the chemoreceptor trigger zone by low-dose opioids, which causes vomiting [18]. In contrast, stronger opioid doses may prevent vomiting by suppressing deeper medulla receptor sites, as seen with midazolam. Being more lipid soluble makes fentanyl more potent and increases its affinity for binding to receptors, which exist almost everywhere in the central nervous system, including in the nucleus of the solitary tract, and, which, when activated, have an antiemetic effect [18]. As a result of this justification, potential antiemetic effect of fentanyl has been established. Observations of higher pruritis with fentanyl have been suggested by Modir et al. [13] , Mehta et al. [11], and Panwar and Chittora [9].

Intrathecal fentanyl with bupivacaine lead to a significantly low pain score in the post-operative period than intrathecal midazolam + bupivacaine. The post-operative analgesic effect was better in group F in comparison to group M (p < 0.001). Several researchers have compared these two as additives to bupivacaine to evaluate the post-operative analgesic effects of the two. The findings of present study are resonated by Ambuj et al. [8], Sawhney et al. [18], Panwar and Chittora [9], and Bhure et al. [10], who suggested that fentanyl had better post-operative analgesia as compared to midazolam as adjuvant intrathecally. This may be because opioid receptors are found in abundance than GABA receptors in the spinal cord. However, Modir et al. [13] found that there is no suggestive difference in group M and group F regarding post-operative pain. On the other hand, Abdelrady et al. [19] reported that median the VAS scores were lesser in midazolam than fentanyl groups.

In the present study, the mean duration of post-operative analgesia was greater with statistical significance in group F, lasting for 367.73 minutes as compared to 254.9 minutes in group M (p < 0.001). Controlling post-operative pain may enable early recovery and discharge from health care facility and enhance the woman's tolerance for early ambulation [14]. The spinal cord modulates and processes nociceptive stimuli. The unearthing of opioid receptors in the spinal cord have led to the use of intrathecal opioids as a method of controlling post-operative pain. Hence, midazolam and fentanyl have been studied as additives by various studies to evaluate the duration of post-operative analgesia. 

The present study found that intrathecal fentanyl as adjuvant leads to prolongation of the duration of post-operative analgesia in comparison to intrathecal midazolam, with corroboration from reports by Ambuj et al. [8], Sawhney et al. [18], Yektas [12], Suraj et al. [16], and Bhure et al [10].

Opioids have the ability to block the ascending transmission of nociceptive-information from the dorsal horn of the spinal cord directly and to activate the pain control circuits that descend from the midbrain to the dorsal horn of the spinal cord via the rostral ventro-medial medulla. This ability gives opioids their analgesic effect. In terms of analgesic potency, fentanyl, a lipophilic opioid, is 100 times more powerful than morphine. It effectively causes altered pain perception and inhibits pain pathways efficiently to provide intra- as well as post-surgical analgesic effect, which gives enhanced satisfaction to the patient [14].

## Conclusions

In this randomized comparative study, we found that intrathecal fentanyl (25 mcg) as an additive to 0.5% bupivacaine (hyperbaric) leads to prolongation of the length of post-operative analgesia significantly as compared to intrathecal midazolam 0.5 mL (2.5 mcg) in women undergoing operative procedure of total hysterectomy by abdominal route under spinal anesthesia. Both intrathecal midazolam and fentanyl have similar efficacy in onset and duration of sensory-motor blockade when used as adjuvants to 0.5% bupivacaine (hyperbaric). The two-segment regression was effectively low when fentanyl was provided intrathecally in comparison to midazolam. Intrathecal fentanyl or midazolam has no significant effects on vital parameters such as HR, RR, systolic blood pressure, diastolic blood pressure, MAP, SPO_2_, and electrocardiography. We conclude that both fentanyl and midazolam are safe for use as adjuvants to 0.5% bupivacaine (hyperbaric) for lower abdominal surgery with minimal side effects.
